# Uncommon Presentation of Granulomatosis with Polyangiitis Mimicking Metastatic Lung Cancer

**DOI:** 10.3390/clinpract11020042

**Published:** 2021-05-14

**Authors:** Edyta Maria Urbanska, Johanna Elversang, Bonnie Colville-Ebeling, Johan Olof Löfgren, Karl Emil Nelveg-Kristensen, Wladimir M. Szpirt

**Affiliations:** 1 Department of Oncology, Rigshospitalet, University of Copenhagen, 2100 Copenhagen, Denmark; 2 Department of Pathology, Rigshospitalet, University of Copenhagen, 2100 Copenhagen, Denmark; johanna.elversang.01@regionh.dk (J.E.); bonnie.colville-ebeling.01@regionh.dk (B.C.-E.); 3 Department of Clinical Physiology, Nuclear Medicine & PET, Rigshospitalet, University of Copenhagen, 2100 Copenhagen, Denmark; johan.olof.loefgren@regionh.dk; 4 Department of Nephrology, Rigshospitalet, University of Copenhagen, 2100 Copenhagen, Denmark; karl.emil.nelveg-kristensen.02@regionh.dk

**Keywords:** intrathoracic lesions, granulomatosis with polyangiitis, GPA, vasculitis, lung cancer differential diagnosis

## Abstract

Diagnosis of anomalous intrathoracic lesions may be challenging and require a multidisciplinary approach. We present a case of granulomatosis with polyangiitis (GPA) clinically and radiologically mimicking metastatic lung cancer with a bilateral pulmonary mass, mediastinal and cervical lymph node involvement, and pleural effusion. Surgical biopsy of the thoracic lesion revealed necrotic granulomatous inflammation, and the final diagnosis was subsequently confirmed by kidney biopsy and biochemical parameters. This case illustrates how comprehensive diagnosis secures timely and relevant treatment. Systemic vasculitis may be one of the key differential diagnoses in patients with multiorgan involvement, especially with pattern-mimicking lung cancer.

## 1. Introduction

Differential diagnosis of abnormal thoracic lesions constitutes an important part of routine clinical work-up and can include a wide spectrum of diseases. As soon as standard diagnostic procedures fail to recognize a patient’s diagnosis, a collaborative approach is required to secure timely and relevant treatment. 

Granulomatous diseases can easily mimic a malignant phenotype as in the current case of granulomatosis with polyangiitis (GPA, Wegener’s granulomatosis). GPA, microscopic polyangiitis (MPA), and eosinophilic granulomatosis with polyangiitis (EGPA) are defined as small-vessel vasculitides associated with antineutrophil cytoplasmic antibodies (ANCAs) [1]. ANCA-associated vasculitis (AAV) is characterized by inflammation of small vessel walls with preferential involvement of the upper and lower airways as well as of the kidneys. However, it can essentially affect any part of the body [2,3,4]. AAV carries a significant risk of mortality and morbidity notwithstanding adequate treatment, and the time from initial symptoms to actual diagnosis is positively correlated with the disease outcome [5,6]. GPA and MPA are rare diseases with a collective incidence of approximately 20 cases per million per year [7]; they are characterized by similar renal histopathological lesions [8] and share a putative ANCA-associated pathogenesis [9]; however, their associated ANCA serology (anti-proteinase 3 and anti-myeloperoxidase) differs in genetic predisposition [10], treatment response, risk of relapse [11], and prognosis [12,13]. Due to the granulomatous phenotype including pseudotumors and lung granulomas as well as the frequently reported longstanding prodromal constitutional symptoms, the diagnosis of GPA often represents a diagnostic challenge which may delay adequate treatment with a subsequent risk of more severe chronic tissue damage, morbidity, and mortality. Accordingly, this case report highlights these diagnostic challenges from the oncologist’s perspective and emphasizes possible pitfalls associated with contemporary standard radiological cancer screening.

## 2. Materials and Methods

**Figure 1:**
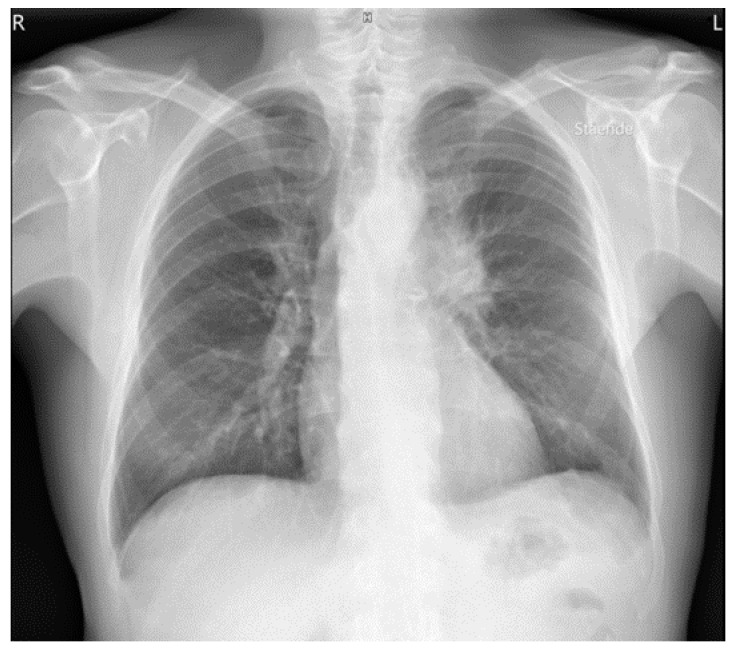
Chest X-ray with a large left hilar mass.

**Figure 2:**
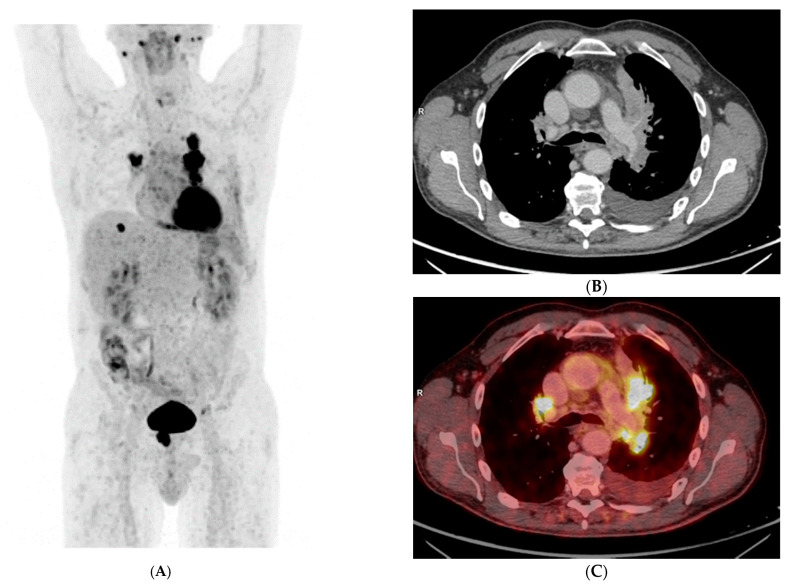
(**A**) Maximum Intensity Projection (MIP) FDG-PET showing multiple FDG-avid lesions, (**B**) transaxial CT image showing a tumor atelectasis complex in the left upper lung’s lobe and enlarged lymph nodes in both hila, (**C**) same as **(B)** fused with FDG-PET.

A 56-year-old Caucasian male, heavy smoker, without known comorbidities, was referred to the Department of Pulmonology with suspected metastatic lung tumor. During the preceding six months, he had experienced increasing shortness of breath, productive cough, and occasional hemoptysis. He also complained of peripheral edema, muscle and joint pain, fever, and unintentional weight loss. An initial chest X-ray showed a left hilar mass (Figure 1), and subsequent fluorodeoxyglucose (^18^F) positron emission tomography–computed tomography (FDG-PET/CT) revealed an 8-cm FDG-avid tumor atelectasis complex suggestive of malignancy in the left upper lobe with mediastinal involvement. The FDG-PET/CT scan also revealed FDG-avid enlarged lymph nodes on the left side of the neck, in the mediastinum at positions 4 L and 7 and at both hila, as well as bilateral pulmonary nodules suggestive of metastases, pleural metastasis in the middle right lung’s lobe, left-sided pleural effusion, a small FDG-avid lesion in both parotid glands and a 3-cm FDG-avid lesion in the prostate (Figure 2A–C, maximum intensity projection (MIP)).

**Figure 3:**
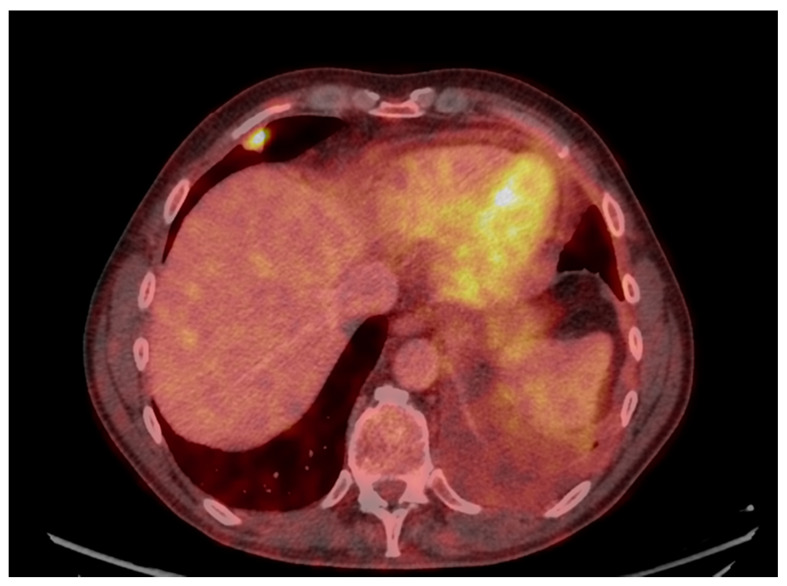
FDG-PET/CT showing pleural metastasis in the right lung’s middle lobe.

Laboratory results showed an active urine sediment with proteinuria (5.48 g/day) and hematuria together with elevated serum creatinine (173 μmol/L). Prostate-specific antigen (PSA), calcitonin, anti-neutrophil cytoplasmic antibodies (ANCA), myeloperoxidase (MPO)-ANCA, anti-glomerular basal membrane (GBM), and immunoglobulins were all normal, but C-reactive protein (CRP) (70 mg/L), proteinase 3 (PR3)-ANCA (44 kU/L), urine albumin creatinine ratio (UACR) (2730 U), blood pressure (172/98 mmHg) were elevated. Serum albumin was low, 21 g/L. Standard additional diagnostic procedures including bronchoscopy with endobronchial ultrasound (EBUS), bronchoalveolar lavage (BAL) and cytological examination of pleural effusion did not show any signs of malignancy. Therefore, to finally confirm or refute cancer, video-assisted thoracoscopic surgery (VATS) with biopsy from the pulmonal tumor in the right middle lung’s lobe was performed (Figure 3), and four days later, because of the coexisting and abovementioned abnormal renal parameters, renal biopsy was performed.

## 3. Results

**Figure 4:**
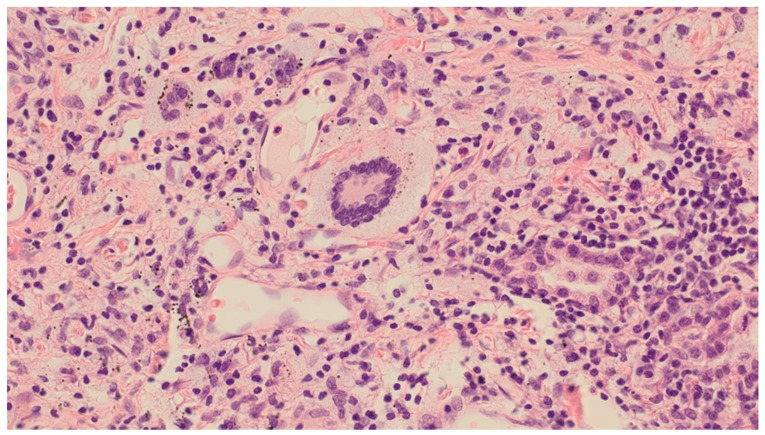
Video-Assisted Thoracoscopic Surgery (VATS) biopsy from the right lung’s middle lobe: Granulomatosis with Polyangiitis (GPA) with a multicore giant cell: a characteristic multinucleated giant cell with smudged basophilic nuclei marginated at the periphery of the cell. Magnification: ×40. Haematoxylin and Eosin (HE) stain.

**Figure 5:**
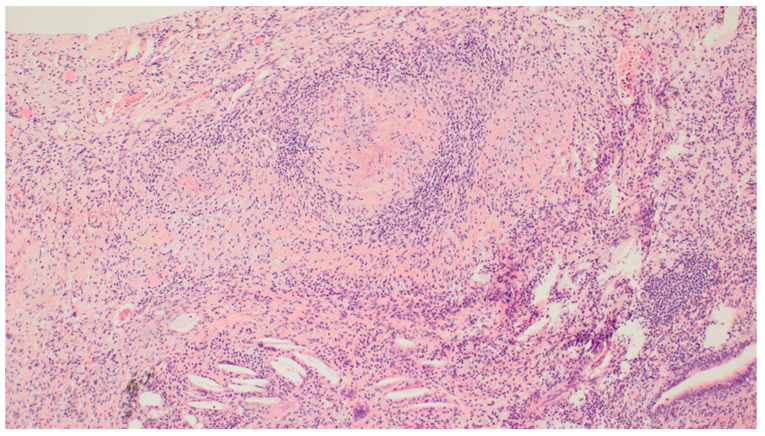
VATS biopsy from the right lung’s middle lobe: GPA blood vessels with vasculitis: destruction of larger vessel walls and lumina due to inflammation. Magnification: ×10. HE stain.

**Figure 6:**
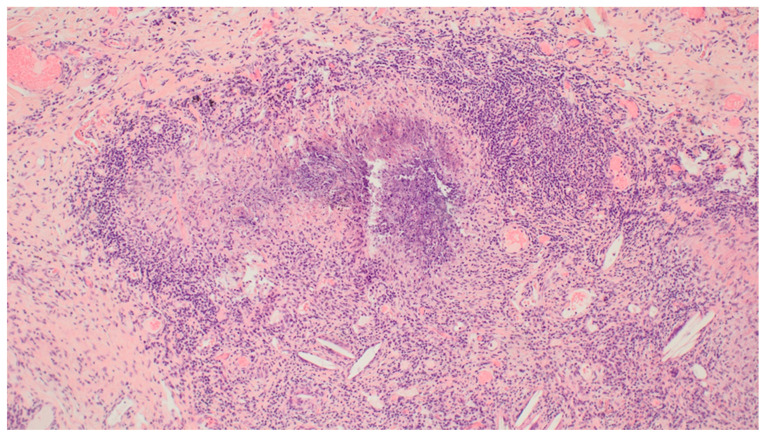
VATS biopsy from the right lung’s middle lobe: GPA necrotizing granuloma: necrotizing granulomatous inflammation and cholesterol clefts. The necrosis is basophilic, with geographical morphology. Magnification: ×10. HE stain.

**Figure 7:**
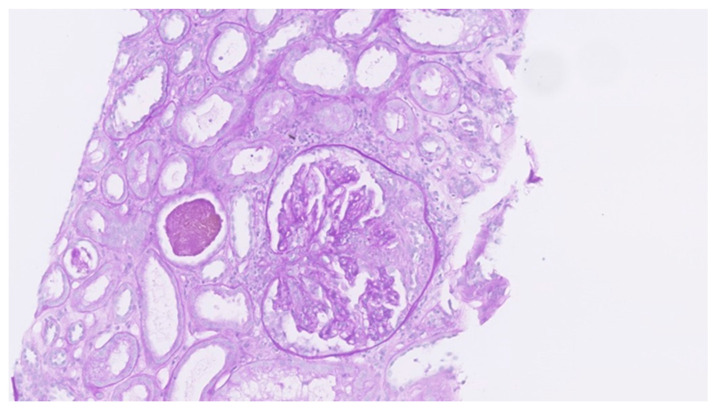
Left kidney: the biopsy showed crescentic glomerulonephritis with focal fibrinoid necrosis of the glomerular tufts end extracapillary proliferation in five out of seven glomeruli. Immunofluorescence was negative and consistent with pauci-immune ANCA-associated glomerulonephritis.

**Figure 8:**
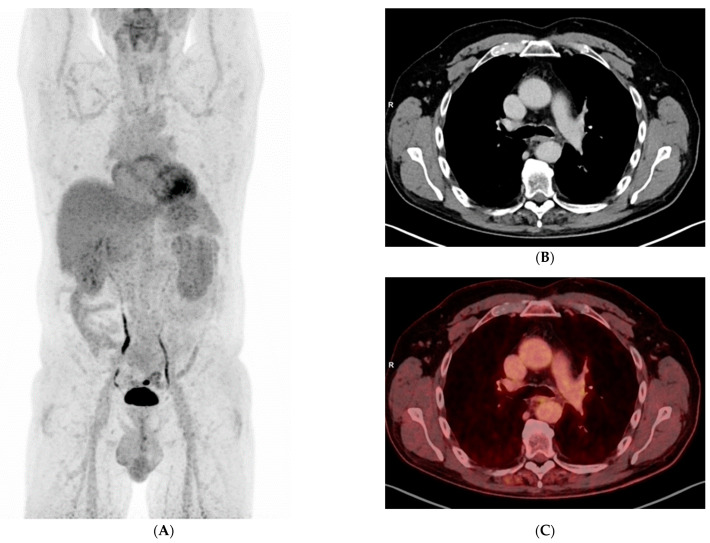
(**A**) MIP FDG-PET showing complete resolution of all the prior FDG-avid lesions, (**B**) transaxial CT image showing resolution of the prior tumor atelectasis complex, (**C**) same as (**B**) fused with FDG-PET.

Histological examination of the formalin-fixed and paraffin-embedded tissue from the right lung’s middle lobe revealed a nodular lesion consisting of a consolidated alveolar parenchyma with necrosis, granulomatous inflammation, multinucleated giant cells, often with smudged basophilic nuclei marginated at the periphery of the cell, and cholesterol clefts (Figure 4, Figure 5 and Figure 6). The necrotic areas were big and confluent focally with geographical morphology and basophilic staining of debris from neutrophil granulocytes as well as palisading histiocytes along the brim. There were scattered microabscesses. The inflammatory infiltrate was dominated by lymphocytes and, to a lesser extent, by plasma cells, eosinophils, and neutrophil granulocytes. There was a little focus of possible capillaritis, but no evident inflammation or fibrinoid necrosis in the vessel walls. However, several of the larger vessels were involved including granulomatous inflammation with destruction of vessel walls and lumina, respectively. Finally, there was a small area of xanthogranulomatous inflammation with alveoli filled with foamy macrophages. The inflammation was seen in close relation to bronchi, but there were no bronchiolitis obliterans, acute bronchiolitis, bronchocentric granulomatosis, or stenosis. No foreign material was identified. Special stains for fungi and mycobacteria were negative, and there were no signs of malignancy. The changes themselves were not pathognomonic for ANCA-associated vasculitis (AAV). However, in lieu of the clinical presentation with joint pain, malaise, and constitutional symptoms, absence of malignancy in EBUS, BAL, cytological examination of pleural effusion, and VATS in association with elevated PR3-ANCA and kidney biopsy showing pauci-immune extracapillary necrotizing glomerulonephritis (Figure 7), the entire picture was consistent with GPA. The patient immediately started the standard treatment regimen for AAV with prednisolone (1 g/kg/day) in combination with oral cyclophosphamide (100 mg/day), and the symptoms subsequently resolved with normalization of biochemical parameters. At follow-up, three months later, FDG-PET/CT showed complete resolution of all the prior FDG-avid lesions (Figure 8A–C). Presently, at 14 months post-discharge, the patient is stable without extrarenal AAV activity, his creatinine has normalized to 81 µmol/L, creatinine clearance—to 91 mL/min, and proteinuria—to 2–3 g/day on the azathioprine and prednisolone maintenance therapy.

## 4. Discussion

GPA and microscopic polyangiitis (MPA) confine the major subgroups of AAV, which represents a complex autoimmune disease of multifactorial etiology that can affect all organ systems with preferential involvement of the skin, respiratory tract, and kidneys. Pulmonary involvement in AAV is a frequent finding and with varying radiographic presentations, i.e., multiple lung noduli of varying size, solid and often cavitary lesions or diffuse ground-glass opacities and fibrosis [14,15]. The current case, however, was exceptional in comparison with many previously described pulmonary presentations as the FDG-PET/CT scan, in addition to lung lesions, identified enlarged thoracic lymph nodes in both hila, mediastinum, neck and pleural effusion. Furthermore, FDG-avid lesions were observed in the prostate and, to some extent, in the parotid glands. The spread pattern was very suggestive of metastatic lung cancer, which was subsequently ruled out by tissue biopsies. However, despite a tentative malignant diagnosis based on the initial radiological findings, standard diagnostic procedures with EBUS and cytological examination of pleural effusion performed in this case failed to confirm cancer diagnosis. This was rather unusual considering the extent and localization of the thoracic lesions. A small FDG-avid lymph node on the neck is often a nonspecific finding and, in case we need to rule out a malignant disease, biopsy from primary thoracic lesions with more clear malignant features is preferred to avoid the risk of prolonging the diagnostic process. At the same time, FDG-avid lesions were also seen in both parotid glands, which often and in most cases represent benign Warthin’s tumors. Therefore, surgical biopsy, which is otherwise not a standard diagnostic procedure, was necessary to provide sufficient tissue to determine histological diagnosis and rule out malignancy. This case report contributes to the previously described importance of surgical biopsy in patients with multiorgan involvement where the ultimate diagnosis is difficult to settle [16]. Evaluating the PET/CT description in the context of metastatic lung cancer, one would expect the patient to be more clinically affected than described. Our case report also confirms the previously reported findings that FDG-PET/CT cannot differentiate between malignant and inflammatory lesions in GPA [17]. Although such radiographical findings might be more indicative of malignancy, the current case, however, underscores the importance of tissue biopsy-based diagnosis with a broad differential diagnostic approach in the initial work-up in patients suspected to have a malignant disease. As reported in a series of 87 open-lung biopsies revealing GPA, 72% had concomitant renal involvement [18], as also confirmed in our case. A hallmark of GPA is the presence of granulomatous inflammation; however, granulomas rarely appear in kidney biopsies in which GPA and MPA share the same lesions. In our lung biopsy, there were no signs of malignancy; however, the findings were not pathognomonic for GPA either as several differential diagnoses can cause necrotizing granulomatous inflammation in the lungs. Accordingly, renal biopsy was performed without further delay revealing pauci-immune extracapillary necrotizing glomerulonephritis consistent with ANCA-associated glomerulonephritis. Thus, considering the clinical, pathological, and biochemical findings, the indisputable diagnosis of GPA was finally settled and the standard treatment with a steroid and cyclophosphamide was initiated and the patient responded with the total remission of all the parameters and today has an almost normal renal function with the estimated glomerular filtration rate (eGFR) of 91 mL/min.

Differential diagnosis in AAV patients includes malignancies (both solid tumors and hematological malignancies), infectious diseases, e.g., pneumonia and tuberculosis, and autoimmune diseases, such as systemic lupus erythematosus (SLE) and sarcoidosis [19,20]. AAV as a paraneoplastic phenomenon seems to be an uncommon presentation of hematological malignancies. However, their coexistence was previously observed, e.g., in a case where initial presentation with cutaneous vasculitis led to the diagnosis of hairy cell leukemia [21,22]. Pulmonary malignancies are not usually reported at AAV diagnosis in patients with pulmonary symptoms. However, a case with probable GPA was reported to be misdiagnosed as lung cancer based on fine-needle aspiration biopsy, and responded to chemotherapy [23]. The authors emphasize though that diagnosis based on needle biopsy may not be sufficient and these two diagnoses might coexist. Conversely, there is an extensive number of malignancy studies during AAV follow-up [24,25,26,27,28]. The course of the disease with frequent relapses can be a challenge as lung tumors can be difficult to distinguish from pulmonary AAV activity [29]. In this study, the authors screened PubMed for relevant publications on AAV and pulmonary malignancies and found six GPA patients with associated lung cancer; only one was diagnosed during the first year of GPA diagnosis. There have been reported cases of AAV with lung cancer within two years after onset and many years later (8–10 years). In a Norwegian cohort of 419 AAV patients, the calculated standardized incidence ratios (SIRs) were 1.09 for all cancer types; however, they were not significantly increased. At the same time, non-melanoma skin cancer (NMSC), posttransplant cancer, and hematologic cancer associated with immunosuppressive treatment were significantly elevated [25]. A meta-analysis of observational studies in AAV patients (six studies with a total of 2578 patients) showed higher pooled SIRs of 1.74 [27]. Lower SIRs than previously were reported when follow-up data from the European Vasculitis Study Group clinical trial were analyzed and compared with previous studies, which was contributed to lower total exposure to cyclophosphamide. Presently, a persistent increased risk of overall malignancy, bladder cancer, and pancreatic cancer, as well as a markedly increased risk of squamous cutaneous skin cancer (SCC), was confirmed [28]. On a positive note, there was no increase in the incidence of cancers other than SCC for those treated with <10 g cyclophosphamide, which is a reference standard for the total cyclophosphamide exposure when used for inductive immunosuppressive treatment in AAV, including in our hospital. 

## 5. Conclusions

Multidisciplinary and cooperative approaches remain paramount in the assessment of abnormal thoracic lesions, including pertinent planning and timing of subsequent diagnostic steps, to facilitate relevant treatment without unnecessary delays. In this case, standard FDG-PET/CT imaging initially gave rise to the suspicion of lung cancer. However, subsequent VATS and kidney biopsy in combination with positive PR3-ANCA refuted malignancy and confirmed the diagnosis of GPA.
